# The Capability of ChatGPT in Predicting and Explaining Common Drug-Drug Interactions

**DOI:** 10.7759/cureus.36272

**Published:** 2023-03-17

**Authors:** Ayesha Juhi, Neha Pipil, Soumya Santra, Shaikat Mondal, Joshil Kumar Behera, Himel Mondal

**Affiliations:** 1 Physiology, All India Institute of Medical Sciences, Deoghar, Deoghar, IND; 2 Pharmacology, All India Institute of Medical Sciences, Bilaspur, Bilaspur, IND; 3 Pharmacology, College of Medicine and JNM Hospital, Kalyani, IND; 4 Physiology, Raiganj Government Medical College and Hospital, Raiganj, IND; 5 Physiology, Dharanidhar Medical College, Keonjhar, Keonjhar, IND

**Keywords:** artificial intelligence, patient education, language model, chatgpt, adverse reactions, side effects, explaining, predicting, drug-drug interaction, drug interactions

## Abstract

Background

Drug-drug interactions (DDIs) can have serious consequences for patient health and well-being. Patients who are taking multiple medications may be at an increased risk of experiencing adverse events or drug toxicity if they are not aware of potential interactions between their medications. Many times, patients self-prescribe medications without knowing DDI.

Objective

The objective is to investigate the effectiveness of ChatGPT, a large language model, in predicting and explaining common DDIs.

Methods

A total of 40 DDIs lists were prepared from previously published literature. This list was used to converse with ChatGPT with a two-stage question. The first question was asked as “can I take X and Y together?” with two drug names. After storing the output, the next question was asked. The second question was asked as “why should I not take X and Y together?” The output was stored for further analysis. The responses were checked by two pharmacologists and the consensus output was categorized as “correct” and “incorrect.” The “correct” ones were further classified as “conclusive” and “inconclusive.” The text was checked for reading ease scores and grades of education required to understand the text. Data were tested by descriptive and inferential statistics.

Results

Among the 40 DDI pairs, one answer was incorrect in the first question. Among correct answers, 19 were conclusive and 20 were inconclusive. For the second question, one answer was wrong. Among correct answers, 17 were conclusive and 22 were inconclusive. The mean Flesch reading ease score was 27.64±10.85 in answers to the first question and 29.35±10.16 in answers to the second question, p = 0.47. The mean Flesh-Kincaid grade level was 15.06±2.79 in answers to the first question and 14.85±1.97 in answers to the second question, p = 0.69. When we compared the reading levels with hypothetical 6th grade, the grades were significantly higher than expected (t = 20.57, p < 0.0001 for first answers and t = 28.43, p < 0.0001 for second answers).

Conclusion

ChatGPT is a partially effective tool for predicting and explaining DDIs. Patients, who may not have immediate access to the healthcare facility for getting information about DDIs, may take help from ChatGPT. However, on several occasions, it may provide incomplete guidance. Further improvement is required for potential usage by patients for getting ideas about DDI.

## Introduction

Drug-drug interactions (DDIs) can have serious consequences for patient health and well-being. Patients who are taking multiple medications may be at an increased risk of experiencing adverse events or drug toxicity if they are not aware of potential interactions between their medications. Therefore, patient education on the risks and consequences of DDIs is essential for promoting safe and effective medication use [1]. Patients should also be advised to keep an up-to-date list of all medications they are taking, including over-the-counter drugs, vitamins, and supplements, and to share this information with their healthcare provider [2]. Additionally, patients should be encouraged to ask questions about their medications and potential interactions with their physicians [3].

In many developing countries, the availability of drugs without a prescription is still being practiced. This encourages self-medication or taking suggestions from non-physician healthcare providers [4,5]. Self-medication may increase the risk of DDIs because individuals may not have the necessary knowledge and expertise to understand the potential risks and side effects of taking multiple medications. There are many online avenues that can provide some guidelines about DDI [6]. However, predicting and understanding DDIs can be challenging due to the complex and multifactorial nature of these interactions [7]. In recent years, there has been a growing interest in leveraging natural language processing (NLP) techniques to aid in DDI prediction and explanation [8].

ChatGPT is a large language model that has been shown to perform well on a variety of NLP tasks, including text classification and question answering. It is being used all over the world. The major advantage of ChatGPT is the capability of generating text in a conversational manner [9]. Previous studies have ascertained the capability of ChatGPT in academic writing [10], in answering various levels of reasoning questions in Pathology and Microbiology [11, 12], or the capability of writing medical examinations [13]. However, no study was conducted to ascertain the capability to predict and explain common DDIs.

In this context, our study aimed to find the effectiveness of ChatGPT in predicting and explaining common DDIs. The study would contribute to the growing body of literature on NLP-based approaches to DDI prediction and explanation. If successful, ChatGPT may be used as a handy tool for aiding clinicians as well as patients in identifying potential drug interactions and making informed decisions.

## Materials and methods

Type and setting

This was a cross-sectional observational study conducted with data that are available for public audit. The primary source of data was the world wide web. The study was conducted from February 20 to March 5, 2023.

Ethics

This study involves the collection of data from an online website and analyzing it. No human or animal was involved in this study. No patient information was used to generate the response from the language model. Hence, this study does not require any ethical committee review.

List of interaction

We have collected the list of DDIs from a previously published article by Kheshti et al. [14]. This article used a total of 40 interaction pairs for testing five software programs. We have used the same set of DDI for checking the capability of ChatGPT.

Data collection

**Figure 1:**
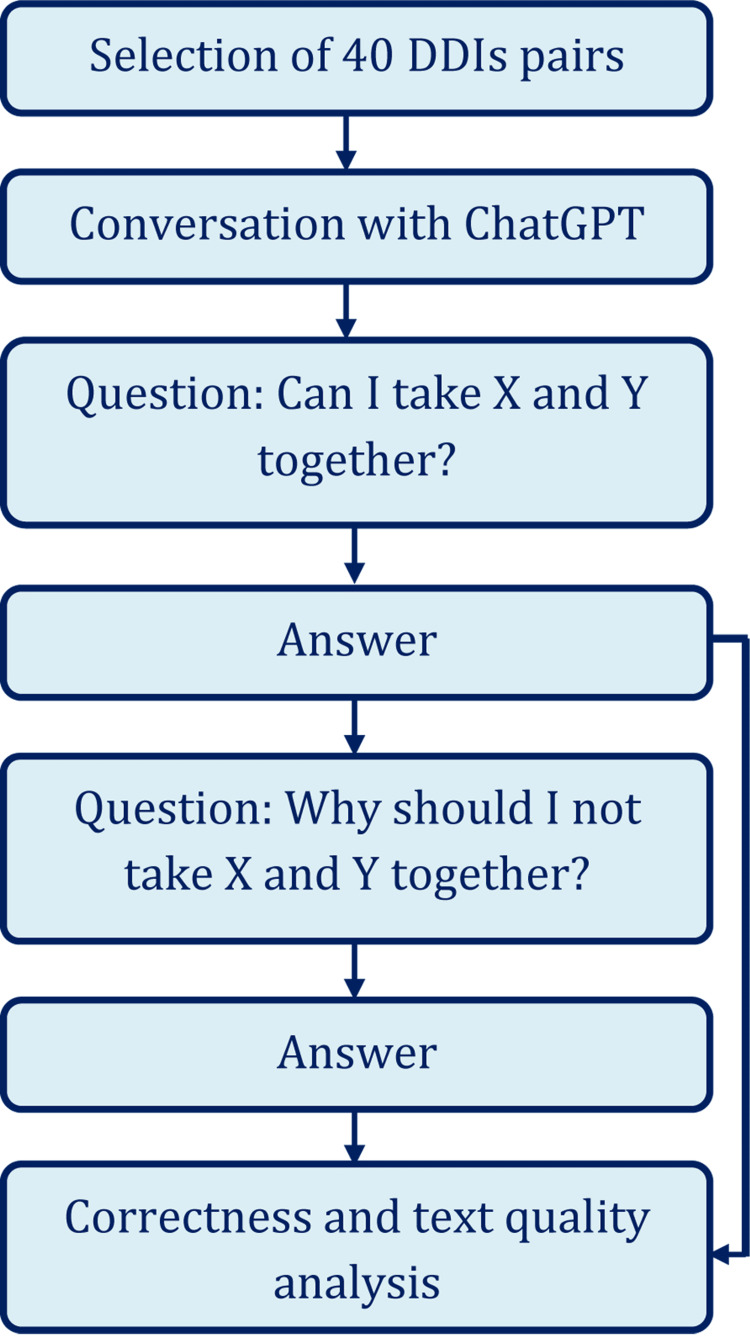
Brief study flowchart DDIs: Drug-drug interactions X and Y were replaced with two drug names during the conversation

A user with a free account in ChatGPT interacted with the artificial intelligence-based language model with the aim to collect data for this study. Each pair of the drugs were searched with two questions - “can I take X and Y together?” and “why should I not take X and Y together?” The output was stored for further analysis. Figure 1 shows the brief process of the data collection method.

Data analysis

The responses were checked by two pharmacologists by taking references from Stockley's Drug Interactions Pocket Companion 2015 and a consensus was reached for categorizing the output as “correct” and “incorrect" [15]. The “correct” ones were further divided into “conclusive” and “inconclusive.” The text was checked for reading ease scores and grades of education required to understand the text. Data were tested by descriptive and inferential statistics. Categorical data were tested by Fisher’s exact test. Continuous variables were tested by unpaired t-test. For checking a single parameter with a hypothetical value, we used a one-sample t-test [16]. For grade level, we considered the American Medical Association and National Institutes of Health to recommend a level that is no higher than a sixth-grade reading (Flesch Reading Ease Score 80-90) [17]. A p-value < 0.05 was considered statistically significant. We used GraphPad Prism (GraphPad Software Inc., USA) to conduct the statistical test.

## Results

Among the 40 DDI pairs, one answer was incorrect in the first question. The rest of the answers were correct, but 19 were conclusive in describing the basis for why the drugs should not be taken together. However, for that one incorrect question, when we asked it “why should I not take X and Y together?”, it rectified the response and informed us that the previous answer was wrongly posted. For the second question, one answer was provided wrong. Among correct answers, 17 were conclusive and the rest were inconclusive. The number of incorrect, correct-conclusive, correct - inconclusive answers is shown in Table 1.

**Table 1:** Correctness of the answer provided by ChatGPT for two types of questions *p-value of Fishers exact test

Category	Correctness		Number	P-value
Answer to “Can I take?”	Incorrect		1	0.003*
	Correct	Conclusive	19	
		Inconclusive	20	
Answer to “Why should I not take?”	Incorrect		1	0.002*
	Correct	Conclusive	17	
		Inconclusive	22	

Output text was analyzed for its sentences, words, syllables per word, reading ease score, and grade levels and we found that the answers to both questions were having equal reading ease scores and require a similar educational grade to understand the text. The words and sentences were higher in the answers to the second question. Text analysis is shown in Table 2.

**Table 2:** Descriptive statistics of generated text output P-values are of unpaired t-test SD: standard deviation, Min: Minimum, Max: maximum *Flesch Reading Ease Score †Flesh-Kincaid Grade Level

Variable	Answer to “Can I take?”		Answer to “Why should I not take?”		P-values
	Mean±SD	Min - Max	Mean±SD	Min - Max	
Sentences	2.85±1.66	1 - 8	3.85±1.87	1 - 9	0.01
Words	59.93±41.27	19 - 198	82.7±38.42	21 - 189	0.01
Word/sentence	21.42±5.59	11.5 – 34.3	21.99±4.22	13.9 – 30.7	0.6
Syllable/word	1.89±0.14	1.6 – 2.2	1.85±0.16	1.5 – 2.3	0.27
Ease score*	27.64±10.85	9.4 – 57.3	29.35±10.16	11.4 – 61.2	0.47
Grade†	15.06±2.79	8.8 – 20.2	14.85±1.97	9.3 – 18.2	0.69

The grade level required for understanding the text was too high for the information to be understood by common users, especially by non-native speakers of English. When we compared the reading levels with hypothetical 6th grade, the grades were significantly higher than expected (t = 20.57, df = 39, p < 0.0001, 95% CI = 8.17 - 9.95 for first answers; t = 28.43, df = 39, 95% CI = 8.22 - 9.48, p < 0.0001 for second answers).

## Discussion

The present study investigated the effectiveness of ChatGPT, a large language model, in predicting and explaining common DDIs. Patients who are taking multiple medications may be at an increased risk of experiencing adverse events or drug toxicity if they are not aware of potential interactions between their medications. ChatGPT was evaluated using a two-stage question format. Although the answers were correct in 39 cases among 40 entries, the correct answers lack clarity in about 50% of the answers. However, this accuracy is measured from a pharmacological point of view. From a layman’s view, the information is correct and can be helpful to detect any DDIs by the patients.

**Figure 2:**
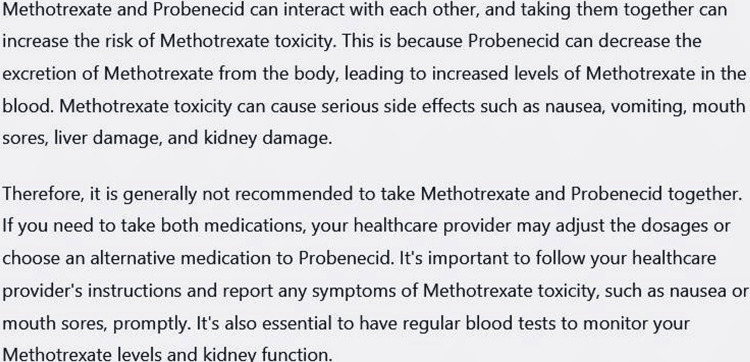
Answer of ChatGPT to question – “Why should I not take Methotrexate and Probenecid together?” The answer was collected on March 10, 2023 and was generated by February 13 version. Flesh-Kincaid grade level 14.1, Flesch reading ease score 27.2

The next issue was about readability. We found that the answers can be understood by a college graduate. This is reasonably higher than the recommended level of grade 6th as suggested by the American Medical Association and National Institutes of Health [17]. Hence, the answer to a direct question about DDI may be difficult to understand for the general population. The underlying reason may be maintaining the scientific contents [18]. However, ChatGPT can be made more productive for documents that are easier to understand. One can instruct the ChatGPT to simplify the language. In response to explaining a DDI, the ChatGPT first provided an output as shown in Figure 2.

**Figure 3:**
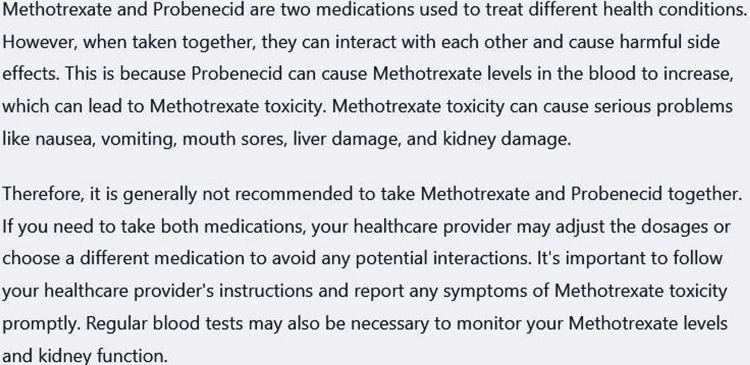
Output of ChatGPT when it was instructed to “explain the above text in lucid language.” Output was collected on March 10, 2023 and was generated by February 13 version. ChatGPT can remember previous conversations with it. Flesh-Kincaid grade level 14.3, Flesch reading ease score 21.3.

We have asked ChatGPT to make the presentation lucid and the output is shown in Figure 3.

**Figure 4:**
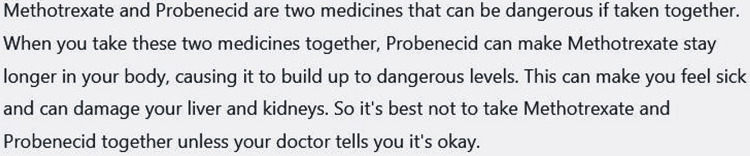
Reply of ChatGPT to the question - “Can you make it simpler so that a 6th-grade student can understand?” Reply was collected on March 10, 2023 and was generated by February 13 version. ChatGPT can remember previous conversations with it. Flesh-Kincaid grade level 9.8, Flesch reading ease score 54.4

However, the grade level did not reduce, and the ease score did not rise. We then asked specifically to make the text suitable for a 6th-grade student and the response is shown in Figure 4.

This time, the grade level was reduced to 10-12th grade with an increment of reading ease score. However, according to the aim, this method of generating text was not practiced and may be considered in further research.

There are several sources of information on DDIs that can help healthcare providers and patients make informed decisions regarding medication use [15]. Drug labels and package inserts provide detailed information on a medication's pharmacology, dosing, and potential drug interactions. Several online tools and mobile applications are available that allow users to check for potential drug interactions between two or more medications. Professional organizations and healthcare institutions often develop guidelines for managing specific medical conditions or prescribing medications. However, it is important to note that information on drug interactions can be complex and may require specialized knowledge. The introduction of ChatGPT is an addition to the already available resources. However, patients and healthcare providers should always consult reliable sources of information and seek advice from a qualified healthcare professional before making any changes to medication regimens.

This study has some limitations. We have used only 40 pairs to test the model and ChatGPT was trained with data that were available till 2021. Hence, the data may not be updated. In addition, the model is regularly being upgraded. Hence, finding at this point in time may not be applicable in the future. We used the free version of the program available at this time and if the capability of the free and paid versions differs, it was beyond our capacity to comment.

## Conclusions

ChatGPT is an artificial intelligence-based model that can help predict and explain DDIs. Patients who do not have immediate access to healthcare facilities can use ChatGPT to get an initial understanding of potential drug interactions. However, ChatGPT may not always provide complete guidance. Patients should always consult with their healthcare provider before starting or stopping any medication, or before making changes to their treatment plan. To make ChatGPT more useful for patients, there is a need for further improvement in its ability to predict and explain DDIs accurately.
